# Optimizing recruitment in rare disease research: a cross-sectional online study evaluating sampling strategies for hard-to-reach populations

**DOI:** 10.1186/s13023-025-04192-3

**Published:** 2026-01-20

**Authors:** Shalice Baffour, Bernd Löwe, Annika Braun, Natalie Uhlenbusch

**Affiliations:** Department of Psychosomatic Medicine and Psychotherapy, University Medical Center Hamburg-Eppendorf, Hamburg, Germany

**Keywords:** Rare diseases, Vulnerable populations, Sampling strategy, Patient recruitment, Medical research, Respondent-driven sampling, Online-based sampling, Location-based sampling

## Abstract

**Background:**

Researchers in the field of rare diseases are often confronted with difficulties in achieving sufficiently large sample sizes, given the small case numbers and geographical dispersion of patients. We aimed to evaluate three different sampling strategies that have proven effective for other *hard-to-reach* populations and compare their effectiveness in the context of rare diseases.

**Methods:**

Within a cross-sectional online study, we compared three sampling strategies (respondent-driven sampling, online-based sampling, and location-based sampling) for their effectiveness in recruiting patients with three rare diseases. Additionally, we compared characteristics of recruited patients. All participants completed an online questionnaire using REDCap.

**Measures:**

Our primary outcome was the number of patients recruited by each sampling strategy. We further assessed study perception, sociodemographic and clinical characteristics, as well as validated measures to assess depression severity (PHQ-9), anxiety severity (GAD-7), illness cognitions (ICQ), health-related quality of life (SF-12) and psychological burden through somatic symptoms (SSD-12).

**Results:**

A total of *N* = 254 individuals accessed the survey website and *N* = 225 completed the sociodemographic characteristics and were included in the analysis. Mean age was 42.53 years (*SD* = 13.06) and *N* = 156 (69%) participants were female. Online-based sampling yielded the highest number of participants (*N* = 184, 82% (95% CI [79%, 85%])), followed by location-based sampling (*N* = 22, 10% (95% CI [4%, 16%])) and respondent-driven sampling (*N* = 19, 8% (95% CI [2%, 14%])). Patient characteristics differed significantly regarding gender and satisfaction with medical care, with online-sampling having the highest share of female participants and patients recruited via location-based sampling reporting a higher satisfaction with their overall care. Across all three sampling strategies, participants showed typical features of populations affected by rare diseases such as high rates of depression and anxiety symptoms and reduced quality of life.

**Conclusions:**

Our study identified online-based sampling as the most effective recruitment strategy for patients with rare diseases. It may be the most promising approach, especially with limited recruitment periods. Potential biases such as gender imbalances should be considered. We encountered substantial challenges with respondent-driven and location-based sampling. Addressing these challenges in future studies may help to make better use of the potential that lies in these sampling strategies.

**Supplementary Information:**

The online version contains supplementary material available at 10.1186/s13023-025-04192-3.

## Background

Rare diseases represent a diverse group of medical conditions, defined by a prevalence of less than 1:2000. Despite their rarity, collectively, there have been identified over 6000 different rare diseases, impacting over 30 million individuals in Europe [1]. Most rare diseases are genetic and often imply physical and psychological burden affecting patients´ quality of life. The availability and effectiveness of treatment options vary depending on the specificity of the condition, however, a shared characteristic among nearly all is the absence of a cure [2]. Symptoms within the same condition can be very different because of genetic variations and timing of presentations, causing delay of diagnosis or misdiagnosis. Even after receiving a diagnosis, access to regular medical care can be difficult due to limited numbers of outpatient clinics and long distances to those existing [3].

Owing to their low prevalence, many rare diseases remain inadequately understood within both societal and healthcare contexts. Enhancing the circumstances for individuals affected could be achieved by fostering broader societal awareness of these conditions, with a particular emphasis on bolstering knowledge within the healthcare framework [2]. Despite increased funding and growing awareness, research in the field of rare diseases faces many challenges. Due to the strong geographic dispersion, limited number of affected patients and a high number of unverified diagnoses, achieving sufficiently large sample sizes can be difficult [4].

In this context, specific sampling strategies for so-called *hard-to-reach* populations can offer valuable insights. Examples of populations that are considered *hard-to-reach* include ethnic minorities, homeless people or drug users [5, 6]. Similar to patients with rare diseases, these groups are characterized by limited accessibility, which makes research challenging and often leads to a lack of knowledge and understanding. A review synthesized studies on barriers to sampling, participation, and retention of *hard-to-reach* populations in health research and described different sampling strategies which can overcome these barriers. Three strategies that are widely used and efficient are respondent-driven sampling (RDS) online-based sampling (OS), location-based sampling (LBS) [6].

Respondent-driven sampling is a probability sampling strategy and a modification of chain referral sampling. It is a network-based approach, assuming that members of *hard-to-reach* populations are best able to access and recruit through their own peers. The first step is to identify participants, called “seeds”, who initiate the recruitment process by referring other people from their social network. These people also refer their peers to the study, so the recruitment process continues. Participants usually only get a determined number of so-called referral coupons to prevent overrepresentation of highly connected individuals, reduce recruitment clustering due to homophily, and support the statistical validity of population estimates [7, 8].

Online-based sampling recruits participants via virtual social networks and websites. Social media platforms, patient forums, and community websites allow direct access to potential participants within interest-based networks, independent of geographic location. Recruitment typically involves disseminating study invitations, digital flyers, or survey links. This approach enables rapid, cost-effective recruitment across large areas while allowing participants to engage anonymously and with low barriers. Interactive features, such as comments, private messaging, or email contact, facilitate communication between researchers and participants and support informed participation [9–11].

Location-based sampling involves identifying and visiting physical sites where members of the target population are expected to be present, such as clinics, community centers, support group meetings, or public events. Researchers approach eligible individuals on-site and invite them to participate. This method targets samples within ecological contexts relevant to the research question and allows recruitment from locations where the population can be reliably reached [12, 13].

This study sets out to identify effective sampling strategies for patients with rare diseases, investigating the three named sampling strategies in terms of their effectiveness and additionally comparing the characteristics of recruited patients. As a secondary objective, the study explores potential differences in psychological and clinical characteristics across samples recruited via the different strategies. To the best of our knowledge, there are no studies comparing different sampling strategies for *hard-to-reach* populations in the context of rare diseases.

## Methods

### Study design

This cross-sectional online study aimed to investigate sampling strategies for *hard-to-reach populations* on the example of patients with rare diseases, while secondarily examining differences in participant characteristics across recruitment strategies. Three different sampling strategies were implemented to recruit patients for a one-time data collection in an identical time interval between July 1^st^, 2023, and November 30^th^, 2023. The recruitment period was set to five months, which was deemed sufficient to capture recruitment dynamics while remaining feasible given the available resources. Ethics approval was given through the psychological ethics committee of the Center for Psychosocial Medicine Hamburg (LPEK) at the University Medical Center Hamburg-Eppendorf (UKE) in Germany on September 17^th^, 2022 (reference number LPEK-0532).

### Study population

We included patients with rare diseases with a prevalence between 0.05–5:10000, which are represented at the Martin Zeitz Center for Rare Diseases at the UKE in Germany. [14, 15]. To represent the heterogeneity in the overall population of rare diseases and different kinds of physical burdens we chose three different and heterogeneous diseases: Marfan syndrome, Huntington disease and lysosomal storage diseases. This selection aims to reflect the diversity of rare diseases and was intended to capture recruitment experiences and psychosocial characteristics that may be relevant across the broader rare disease population.

Marfan syndrome is a systemic disorder of connective tissue with a prevalence ranging between 1 and 5:10000, characterized by a combination of cardiovascular, muscolo-skeletal, ophthalmic, and pulmonary manifestations. Symptoms can appear at any age, and the prevalence is the same for both genders [16–18]. Huntington disease is a neurodegenerative disorder of the central nervous system marked by uncontrollably choreatic motions, behavioral and psychiatric symptoms and dementia [19]. Prevalence ranges between 1-9:10000 and is the same for both genders, with a mean age at symptom onset from 30-50 years [20]. Lysosomal storage disorders describe a group of over 70 diseases which are characterized by lysosomal dysfunction with an estimated prevalence of 0.05:10000 typically occurring in infancy and childhood [21]. There are gender differences in the prevalence of lysosomal storage diseases. This varies depending on the specific condition due to genetic inheritance patterns [22]. Lysosomal dysfunction leads to an accumulation of undegradable material in lysosomes of different cell types, resulting in neurodegenerative symptoms, skeletal alterations and dysfunction of organs [21].

Inclusion criteria were an age of at least 18 years, the diagnosis of one of the three diseases, sufficient German skills, internet access and online informed consent. We excluded patients under the age of 18 and those who reported having an unconfirmed diagnosis.

### Setting and procedures

We recruited participants using respondent-driven sampling, online-based sampling and location-based sampling. Details for each sampling strategy are described below. To reduce bias, all sampling strategies were implemented at the same time, from 1^st^ July 2023 to 30^st^ November 2023. We designed flyers containing study information and a QR-Code and link directing participants to the online questionnaire. Flyers were uniformly prepared for each sampling strategy and medical condition. Flyers for respondent-driven sampling contained additional instructions on how to distribute flyers to their peers (for more information see next section). While online-based sampling and respondent-driven sampling utilized digital distribution, location-based sampling employed printed flyers for dissemination. To enhance response rates, reminders were issued at four-week intervals for all sampling strategies. The use of unique QR-Codes and links for each sampling strategy allowed participants to be associated with their respective recruitment way. All recruited patients anonymously completed the online questionnaire on REDCap. REDCap is a web-based, secure application created to build and manage online surveys and databases [23, 24].

#### Respondent-driven sampling

To identify suitable initial seeds, we conducted a Google search for self-help groups and patients’ associations related to the defined diseases. All organizations identified were contacted via e-mail or phone call. Our aim was to identify affected individuals who are well connected within their community. Selected seeds were either group leaders themselves or individuals recommended by them, who we then contacted. Each seed was informed about the procedure and their role in the recruitment process through personalized telephone calls or e-mails, and subsequently received online study flyers and links to the questionnaire to share with their peers. In our study design, no physical coupons were used; instead, seeds were asked to forward the study link electronically to a maximum of five potential participants within their network.

#### Online-based sampling

For the online recruitment, Facebook was selected as platform due to its accessibility and the availability of established group-based patient communities. We searched Facebook for groups of the targeted diseases and submitted a membership request to upload information about our study. In case of a rejected request, we contacted group administrators personally to inform them about our study and asked them for support. Inclusion criteria were at least one post in the last twelve months (status: April 2023). To prevent overlaps, we did not include Facebook groups associated with the self-help groups and patient organizations we contacted for respondent-driven sampling. A link leading to the questionnaire and the flyer were uploaded with a short introductory comment about the study.

#### Location-based sampling

We aimed to sample patients at specialized medical centers across Germany, where patients with the three rare diseases receive medical care. We conducted a Google search for outpatient clinics in Germany, contacted all we found in the search, seeking their support in patient recruitment. After thoroughly informing the outpatient clinics via e-mail and phone calls, printed study flyers were provided to physicians and study nurses at the clinics who distributed flyers to patients meeting inclusion criteria.

### Variables

Our primary outcome was the effectiveness of the three sampling strategies, as determined by the number of patients each strategy recruited. To trace the sampling strategy participants were recruited with, we used the link or QR-Code patients used to reach the online questionnaire. We also asked patients where they heard about the study and assessed whether their answer is consistent with the survey link, they opened. To address the second objective and examine potential differences in patient characteristics across sampling strategies, we collected a set of variables encompassing sociodemographic and clinical characteristics, as well as key aspects of mental health and psychosocial well-being in patients with rare diseases [25]:

#### Sociodemographic characteristics

Sociodemographic data included gender, age, and employment status and were assessed with single items.

#### Study perception

To assess participants’ subjective perception of the study, we utilized questions on the importance, trustworthiness, beneficialness, and seriousness of the study using numeric rating scales (1 = not important/trustworthy/beneficial/serious at all, 10 = greatest imaginable importance/trustworthiness/beneficialness/serosity).

#### Clinical characteristics

Clinical data was assessed as a descriptive characteristic to contextualize differences in participant profiles across recruitment strategies. It included diagnosis, time since symptom onset and diagnosis, time to find specialized medical care, journey duration to specialized medical care and contentment with medical care. Time specifications were assessed with dates, diagnoses were assessed with categorical questions and contentment with medical care was assessed with a numeric rating scale (1–10).

#### Depression symptom severity

The Patient Health Questionnaire (PHQ) is a self-administered diagnostic tool based on the PRIME-MD (Primary Care Evaluation of Mental Disorders) instrument, designed to assess mental disorders. The PHQ-9 serves as the depression module, evaluating each of the 9 DSM-IV criteria on a scale from 0 to 3. The sum-score can indicate symptom severity of depression. As a singular threshold indicating elevated depression severity, the authors propose a sum-score of ≥ 10 [26]. International research has confirmed the robustness of its psychometric properties. A thoroughly validated German version of the PHQ-9 is available [27–29].

#### Anxiety symptom severity

The German version of Generalized Anxiety Disorder 7-item scale (GAD-7) was used to assess symptom severity of generalized anxiety disorders and other anxiety disorders [30–32]. Symptoms are evaluated with a sum score, for which a value ≥ 10 indicates elevated anxiety severity [30]. Its psychometric properties include strong internal consistency [31], good test-retest reliability and demonstrated validity in terms of criterion, construct and factorial validity as well as procedural aspects [30].

#### Illness cognitions

The Illness Cognition Questionnaire (ICQ) is a self-report tool designed to evaluate patients´ cognitive assessment of their illness. Comprising three subscales (helplessness, acceptance, and perceived benefits), each containing six items, the ICQ generates sum scores ranging from 0 to 18 for each scale [33]. Participants respond to each item using a four-point Likert scale to express their level of agreement with the item [34]. The questionnaire has undergone validation across diverse patient samples, demonstrating favorable concurrent validity, strong internal consistency, and high test-retest reliability [33, 34].

#### Health-related quality of life

We used the German version [35] of the SF-12 health survey to assess health-related quality of life. The SF-12 uses twelve items to measure mental (MCS) and physical aspects (PCS) of generic, health-related quality of life forming sum scores. Scales range for the PCS and MCS were standardized for the 2009 U.S. general population with a mean (*M*) of 50 and a standard deviation (*SD*) of 10 [36, 37]. Developed from the original SF-36, the SF-12 has demonstrated robust psychometric properties through several studies [36, 38, 39].

#### Psychological burden related to somatic symptoms

The Somatic Symptom Disorder B Criteria Scale 12 (SSD-12) assesses the psychological criteria of DSM-5 somatic symptom disorder [40]. Consisting of twelve items, each of the three psychological sub-criteria (affective, cognitive, and behavioral aspects) is represented by four items, with scores ranging from 0 to 4. It has sound psychometric properties [41, 42] and norm values have been derived from a large sample of the German population, enabling comparisons of SSD-12 scores with representative data. Symptoms are evaluated with a sum score, for which a value ≥ 23 indicates high psychological burden related to somatic symptoms [41]. The development of the SSD-12, which is available in many languages, took place in Germany [40].

### Sample size

We did not implement an a-priori sample size determination, because the study aimed to investigate how many people can be recruited with each sampling strategy in a fixed time. The results of this study may help to determine realistic sample sizes of future studies, which include patients with rare diseases.

### Data analysis

Participants who completed at least the sociodemographic characteristics were included in data analysis. We did not perform data imputation for missing data; only complete data was included in the analysis. We computed means and standard deviations for continuous variables, and frequencies and valid percentages for categorical data. We determined the percentage of patients screening positive for a depressive disorder (PHQ-9), an anxiety disorder (GAD-7) or psychological burden through somatic symptoms (SSD-12), by creating binary variables where 1 indicates being above the cut-off and 0 indicates being below the cut-off. One-way ANOVA was considered as a parametric test to compare outcomes between the different groups based on the sampling strategies under the assumptions of independent measurement, a nominal-scaled independent variable and an interval-scaled dependent variable. Additionally, dependent variables were required to demonstrate normal distribution (Shapiro-Wilk test) and homogeneity of variance (Levene’s test). In cases of assumption violations, Kruskal-Wallis test was considered as a non-parametric alternative for metric variables. For categorical variables, Chi-Square/Fisher’s exact test was conducted accordingly. Potential post-hoc procedures were Tukey for one-way ANOVA, Dunn’s test for Kruskal-Wallis test and z-tests with Bonferroni adjustment for Chi-Square test, following recommendations by Field (2013) [43]. Categories with case numbers of *n* < 5 were excluded from statistical analysis. All analyses were performed two-sided with a significance level set at *p* < 0.05 using IBM SPSS 29.

## Results

### Recruitment process

The number of self-help groups, outpatient clinics and social media groups supporting the recruitment differed widely between the three diseases. The exact distribution can be seen in the additional files (Additional file 1).

#### Respondent-driven sampling

Forty self-help groups and patients’ associations were contacted to assist in identifying eligible seeds. We received answers from 12 self-help groups, through which we were able to recruit 11 seeds participating in the study. We could not recruit any seeds with Huntington disease.

#### Online-based sampling

On Facebook, we found 11 online groups, which met our inclusion criteria. Membership requests were accepted by 6 groups. Due to privacy concerns about the medical data shared by group members, some administrators did not want to accept our request but offered to upload the link and flyer. This was the case for 3 groups. Our request was not accepted by 2 groups.

#### Location-based sampling

We contacted 65 specialized outpatient clinics. In total, we received 32 answers, from whom 14 supported us with patient recruitment.

### Sociodemographic characteristics

Gender ratios differed between sampling strategies (*p* = 0.022) with more females being recruited via online-based sampling compared to location-based sampling and more males being recruited via location-based sampling compared to online-based sampling. Non-binary individuals or those who did not wish to provide details were excluded from the statistical analysis due to low case numbers (*n* < 5). There were no further significant differences between the sampling strategies regarding sociodemographic characteristics. Detailed findings are displayed in Table 1.

**Table 1:** Sociodemographic characteristics by sampling strategy

Variable	Total sample	Respondent-driven sampling	Online-based sampling	Location-based sampling	*H* **(df)/χ**^***2***^ **(df)**	p
*N*	225	19	184	22		
**Age**						
mean (SD) median (range)	42.53 (13.06) 41.00 (18–74)	44.11 (15.47) 39.00 (20–71)	42.41 (12.97) 40.00 (18–74)	42.23 (12.10) 43.50 (24–58)	*H*(2) = 0.02	0.989
*N*	225	19	184	22		
**Gender**	*N* (%)	*N* (%)	*N* (%)	*N* (%)		
female	156 (69%)	12 (63%) _a,b_	134 (73%) _a_	10 (46%) _b_	*Χ*^*2*^(2) = 7.44	0.022
male	65 (29%)	6 (32%) _a,b_	47 (26%) _a_	12 (55%) _b_		
non-binary^1^	1 (1%)	0	1 (1%)	0		
I do not wish to provide details^1^	3 (1%)	1 (5%)	2 (1%)	0		
*N*	225	19	184	22		
**Employment**	*N* (%)	*N* (%)	*N* (%)	*N* (%)		
employed	117 (52%)	7 (37%)	98 (53%)	12 (55%)	*X*^*2*^(8) = 10.88	0.154
unable to work	42 (19%)	3 (16%)	33 (18%)	6 (27%)		
student/school	16 (7%)	2 (11%)	11 (6%)	3 (14%)		
retired	18 (8%)	4 (21%)	14 (8%)	0		
other	32 (14%)	3 (16%)	28 (15%)	1 (5%)		

*Notes.* ^1^Categories with *n* < 5 were excluded from analysis; Subscript letters indicate whether the column proportions for the different groups differ significantly from each other at the 0.05 level, with a different letter indicating a statistically significant difference. H: Kruskal Wallis, χ2: Chi-square test, post-hoc test: z-tests with Bonferroni adjustment

### Effectiveness of the three sampling strategies

**Fig. 1:**
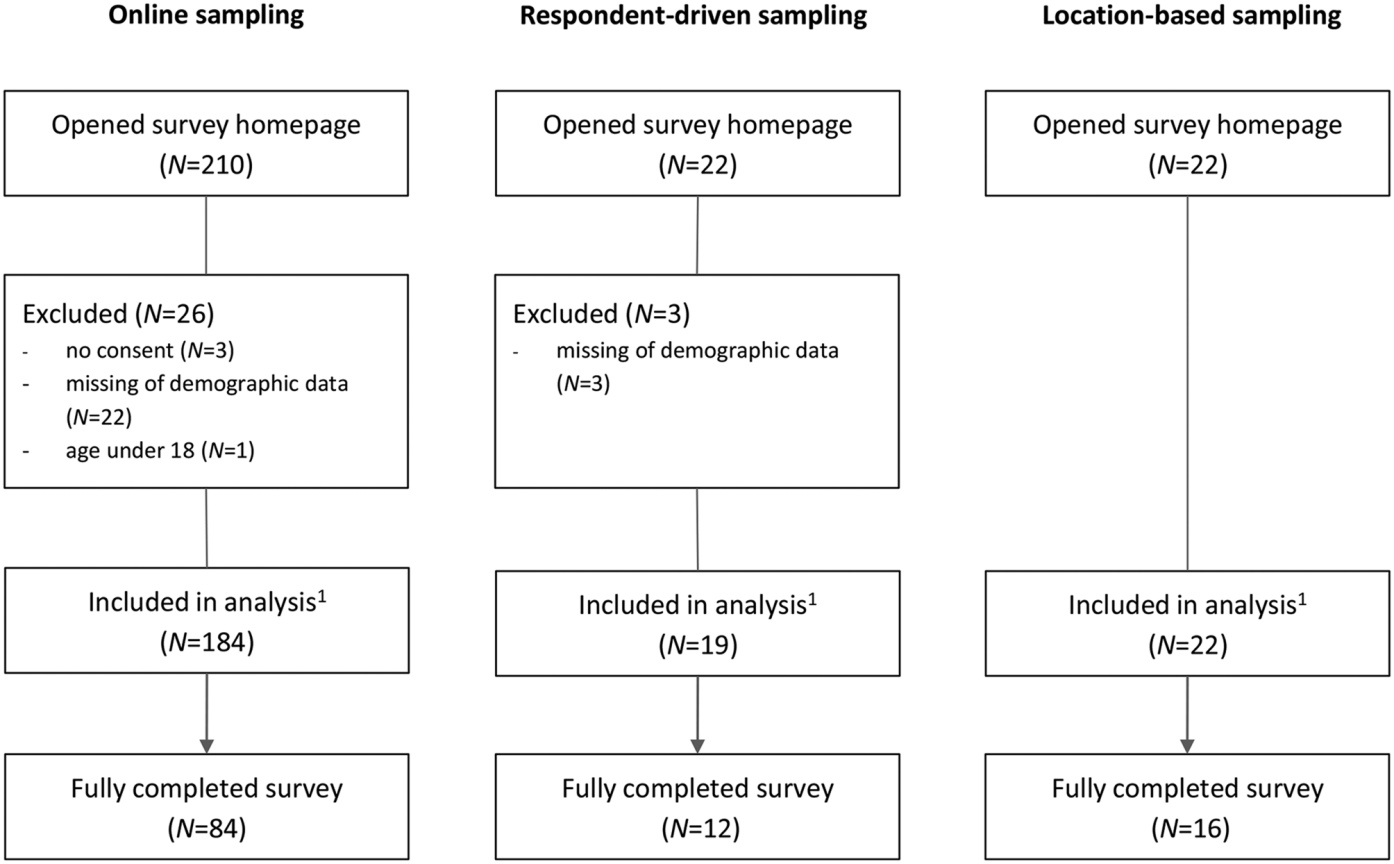
Sample size overview. *Notes.* ^1^ completed at least sociodemographic characteristics

A total of *N* = 254 individuals opened the survey website, from which *N* = 225 (89%) completed the sociodemographic questions and were therefore included in the analysis. Online-based sampling resulted in the highest number of participants (*N* = 184, 82% (95% CI [79%, 85%]), followed by location-based sampling (*N* = 22, 10% (95% CI [4%, 16%]) and respondent-driven sampling (*N* = 19, 8% (95% CI [2%, 14%]. *N* = 112 filled out the survey form completely. The link patients opened was consistent with the self-reported recruitment strategy in nearly all the participants (except one who opened the survey via the online link but self-reported that he was recruited by a person within his social environment). Some patients indicated they heard about the study through different sources. Details of the sampling process are shown in Fig. 1.

### Participant characteristics by sampling strategy

We analyzed whether participants recruited via different sampling strategies differed concerning sociodemographic, clinical, and psychological outcomes as well as their perceptions of the study.

### Study perception

We found no differences between the different sampling strategies. Overall, participants rated the study as important, trustworthy, beneficial, and serious. Results on how patients perceived the study are provided in Table 2.

**Table 2:** Study perception by sampling strategy

Variable	Total sample	Respondent-driven sampling	Online-based sampling	Location-based sampling		
*N*	112	12	84	16		
**Study perception**					***H*** **(df)**	***p***
**Importance**^**1**^ mean (SD) median (range)	7.01 (2.60) 8.00 (0–10)	6.92 (2.35) 8.00 (3–10)	6.96 (2.58) 8.00 (0–10)	7.31 (3.03) 8.00 (1–10)	*H*(2) = 0.82	0.664
**Trustworthiness**^**1**^ mean (SD) median (range)	7.08 (2.67) 8.00 (0–10)	6.83 (2.44) 7.00 (3–10)	6.95 (2.68) 7.50 (0–10)	7.94 (2.77) 9.00 (1–10)	*H*(2) = 3.42	0.181
**Beneficialness**^**1**^ mean (SD) median (range)	6.81 (2.76) 7.00 (0–10)	6.67 (2.31) 7.00 (3–10)	6.93 (2.82) 8.00 (0–10)	6.31 (2.85) 7.00 (1–10)	*H*(2) = 1.18	0.555
**Serosity**^**1**^ mean (SD) median (range)	7.65 (2.49) 8.00 (0–10)	7.83 (2.17) 8.00 (4–10)	7.51 (2.51) 8.00 (0–10)	8.25 (2.62) 10.00 (1–10)	*H*(2) = 2.59	0.273

*Notes.* ^1^rated on numeric rating scales from 1 (not important/trustworthy/beneficial/serious at all) to 10 (greatest imaginable importance/trustworthiness/beneficialness/serosity); H: Kruskal Wallis

### Clinical characteristics

A significantly higher proportion of participants with lysosomal storage disorders (*p* = 0.044) were recruited through respondent-driven sampling compared to online-based sampling. Furthermore, satisfaction regarding medical care was significantly greater in participants recruited via location-based sampling compared to online sampling (*p* = 0.013). Table 3 presents detailed findings.

**Table 3:** Clinical characteristics by sampling strategy

Variable	Total sample	Respondent-driven sampling	Online-based sampling	Location-based sampling	*H* **(df)/** *χ*^*2*^ **(df)**	p
*N*	137	16	103	18		
**Disease**^1^						
Marfan syndrome	64 (47%)	4 (25%)	50 (49%)	10 (56%)	*Χ*^*2*^(2) = 6.28	0.044
Huntington disease	19 (14%)	0	18 (18%)	1 (6%)		
Lysosomal storage dis.	54 (40%)	12 (75%) _b_	35 (34%) _a_	7 (39%) _a,b_		
*N*	124	15	94	15		
**Time between symptom onset and diagnosis** (years)						
mean (SD) median (range)	10.89 (12.89) 4.00 (0–40)	9.67 (7.41) 7 (0–20)	11.03 (13.29) 3.50 (0–43)	11.21 (15.19) 3 (0–39)	*H*(2) = 1.26	0.533
*N*	111	11	86	14		
**Time needed to find specialized medical care** (years)						
mean (SD) median (range)	1.82 (5.80) 0.17 (0–40)	5.73 (10.17) 0.04 (0–30)	0.97 (3.19) 0.17 (0–20)	3.93 (10.79) 0.17 (0–40)	*H*(2) = 0.39	0.823
N	123	15	91	17		
**Travel time to specialized medical care** (hours)						
mean (SD) median (range)	1.79 (1.55) 1.17 (0–7)	2.75 (2.24) 3.33 (0–4.5)	1.69 (1.45) 1.13 (0.1–7)	1.51 (1.06) 1.00 (0.2–2.5)	H(2) = 4.26	0.119
N	137	16	103	18		
**Satisfaction with medical care**^**2**^						
mean (SD) median (range)	6.46 (2.80) 7.00 (1–10)	6.31 (2.36) _a,b_ 6.50 (1–10)	6.19 (2.87) _a_ 7.00 (1–10)	8.11 (2.27) _b_ 9.00 (1–10)	*H*(2) = 8.75	0.013

*Notes*. ^1^Categories with *n* < 5 were excluded from analysis; ^2^rated on numeric rating scales from 1 (not satisfied at all) to 10 (greatest imaginable satisfaction); Subscript letters indicate whether the column proportions for the different groups differ significantly from each other at the 0.05 level, with a different letter indicating a statistically significant difference. H: Kruskal Wallis, post-hoc test: Dunn’s test; χ2: Chi-square test, post-hoc test: z-tests with Bonferroni adjustment

### Psychological characteristics

We found no significant differences between the different sampling strategies regarding depression and anxiety severity, illness cognitions, health-related quality of life, or psychological burden through somatic symptoms. Overall, nearly 50% of the participants showed depressive symptoms above the PHQ-9 cut-off and 32% showed anxiety symptoms above the GAD-7 cut-off, indicating clinically relevant psychopathology. Health-related quality of life sum scores were lower than the standardized sum scores of 50. Details are shown in Table 4.

**Table 4:** Psychological characteristics

Variable	Total sample	Respondent-driven sampling	Online-based sampling	Location-based sampling	*H***(df)/** *χ*^*2*^(**df)**		p
N	118	13	89	16			
**PHQ-9 sum score**							
mean (SD) median (range)	9.89 (6.09) 9.00 (0–24)	9.38 (5.32) 8.00 (4–24)	10.45 (5.99) 10.00 (0–24)	7.19 (6.75) 5.00 (0–21)	*H*(2) = 4.34		0.114
cut-off ≥ 10							
*N* (%)	57 (48%)	5 (39%)	47 (53%)	5 (31%)	*X*^*2*^ (2) = 3.03		0.213
*N*	117	13	88	16			
**GAD-7 sum score**							
mean (SD) median (range)	7.15 (5.13) 6.00 (0–21)	7.00 (5.37) 5.00 (1–16)	7.43 (5.27) 7.00 (0–21)	5.75 (4.07) 5.00 (0–13)	*H*(2) = 1.03		0.598
cut-off ≥ 10							
*N* (%)	37 (32%)	4 (31%)	29 (33%)	4 (25%)	*X*^*2*^ (2) = 0.37		0.943
*N*	115	13	86	16			
**ICQ sum score**							
**helplessness**							
mean (SD) median (range)	13.95 (4.97) 13.00 (6–24)	15.23 (3.79) 14.00 (10–21)	13.88 (4.98) 13.00 (6–24)	13.25 (5.85) 12.50 (6–24)	*H*(2) = 1.49		0.474
**acceptance**							
mean (SD) median (range)	15.46 (4.65) 16.00 (6–24)	14.38 (3.10) 16.00 (7–18)	15.53 (4.84) 16.00 (6–24)	15.94 (4.74) 17.00 (7–23)	*H*(2) = 1.52		0.468
**perceived benefits**							
mean (SD) median (range)	14.95 (5.12) 15.00 (6–24)	13.31 (3.28) 13.00 (9–20)	15.14 (5.33) 15.00 (6–24)	15.25 (5.18) 15.00 (7–24)	*H*(2) = 1.24		0.537
N	121	13	91	17			
**SF-12**							
physical sum score							
mean (SD) median (range)	36.68 (7.52) 36.65 (22–55)	33.04 (8.59) 31.79 (22–48)	37.03 (7.19) 37.10 (24–55)	37.62 (8.05) 38.62 (24–49)	*H*(2) = 2.97		0.227
mental sum score							
mean (SD) median (range)	44.30 (9.09) 44.70 (19–63)	45.51 (6.42) 46.03 (35–56)	43.98 (9.34) 44.15 (19–59)	45.06 (9.74) 48.23 (23–63)	*H*(2) = 0.37		0.832
*N*	113	13	84	16			
**SSD-12 sum score**							
mean (SD) median (range)	22.43 (10.54) 22.00 (0–48)	25.85 (6.73) 26.00 (14–36)	22.52 (10.99) 22.50 (0–48)	19.19 (10.12) 19.50 (2–37)	*H*(2) = 1.03		0.226
cut-off ≥ 23							
*N* (%)	56 (50%)	8 (62%)	42 (50%)	6 (38%)	*X*^*2*^ (2) = 1.66		0.448

*Notes.* H: Kruskal Wallis, χ2: Chi-square test

## Discussion

This study aimed to evaluate three sampling strategies for *hard-to-reach* populations regarding their effectiveness among patients with three different rare diseases. All findings regarding the performance of the different sampling strategies are descriptive, reflecting the number of participants each approach reached. The three sampling strategies were respondent-driven sampling, online sampling, and location-based sampling. Among the three, online sampling yielded the largest sample size, indicating its effectiveness in reaching a high number of participants while at the same time requiring relatively small recruitment efforts in terms of logistics and time investment, compared to respondent-driven sampling and location-based sampling. This is consistent with previous research underscoring the effectiveness of online sampling in studies involving patients with rare diseases [11]. Web-based approaches have also been shown to efficiently reach geographically dispersed patients, particularly through established online communities [44]. However, high reach does not always translate into actual enrollment, as administrative or procedural hurdles can limit recruitment effectiveness [45].

The number of participants recruited via respondent-driven sampling and location-based sampling was relatively low, while in prior studies, both sampling strategies have been successful in recruiting *hard-to-reach* populations in various contexts [46, 47]. For both strategies, we encountered more challenges compared to online-based sampling. In respondent-driven sampling, participants struggled to understand the referral system. Furthermore, some group leaders were skeptical about the effectiveness of respondent-driven sampling, believing that it would not work for their specific contexts, which may have biased recruitment. Additionally, the absence of incentives in our study may have contributed to the limited sample size [48]. Lastly, feedback from self-help groups indicated a decline in physical meetings since the onset of the COVID-19 pandemic in 2020, with some groups not meeting in person for over 3.5 years. This reduction in face-to-face interactions has likely diminished networking opportunities, which may have impeded the distribution of study information. Similar challenges were noted in other respondent-driven sampling studies involving *hard-to-reach* populations, where barriers included low participant motivation due to perceived inadequate incentives, low-density networks, and logistical difficulties [48]. Psychosocial factors, such as high levels of self-stigmatization, may also affect participation in this sampling strategy [49]. In location-based sampling, effective communication with outpatient clinics was challenging due to a lack of consistent contact persons, resulting in slow response times. Some outpatient clinics expressed a willingness to support our study but encountered hurdles related to complex internal ethics approval processes. The low number of participants recruited via location-based sampling may further be attributed to the study’s limited duration of five months. According to several outpatient clinics, extending the study period to one year could have been beneficial, as many patients visit these clinics only once annually and are otherwise under the care of their general practitioners. Taken together, online-sampling not only resulted in the highest number of participants, but the recruitment process was also associated with substantially less challenges compared to the other two sampling strategies.

The characteristics of participants across all three sampling strategies exhibited typical features of populations with rare diseases. The diagnostic delay observed in our study averaged close to 11 years, even exceeding findings from a US study where participants with rare diseases experienced a diagnostic delay of approximately 9 years [50]. Additionally, patients in our study reported travel times to specialized care ranging from 1.51 to 2.75 hours, which is in line with research indicating that large numbers of participants report barriers to accessing medical care, including extended travel times [51]. Results on depression and anxiety severity, illness cognitions, health-related quality of life and psychological burden through somatic symptoms indicate that patients experience high physical and mental burden. Almost half of the participants showed depression symptoms above the cut-off, indicating a clinically relevant depressive disorder and clinically relevant anxiety symptom severity was found in 32%. Half of the participants showed high psychological burden through their somatic symptoms. These outcomes are consistent with previous research addressing challenges faced by individuals with rare diseases [2, 15] and confirm the high physical and mental burden of the rare disease community across conditions.

We observed a few statistically significant differences between patients recruited by different sampling strategies. Notably, online-based sampling included a higher proportion of women compared to respondent-driven and location-based sampling. While these differences describe patterns within our study sample rather than population-level effects, they may reflect factors such as gender differences in social media use [52], which could have contributed to higher participation of women in online recruitment. Additionally, studies indicate that women with rare diseases might exhibit a higher demand for information, leading them to engage more with online health-related content [53, 54]. Furthermore, satisfaction with medical care was rated significantly higher among patients recruited via location-based sampling compared to those from respondent-driven and online sampling. As location-based sampling was conducted at specialized outpatient clinics, the higher satisfaction may reflect better access to adequate care. We found no further statistically significant differences between the sampling strategies.

## Limitations

The population was highly heterogeneous, which, along with the small sample size, made the consideration of disease-specific differences unfeasible. This heterogeneity corresponds to the overall population of rare diseases, which is why three diseases with varying clinical manifestations were selected. Still, it is unclear to what extent these three conditions represent the overall population of 7000 different rare diseases. All data were self-reported, introducing potential recall bias, particularly for variables related to past events, such as the time of diagnosis. While well-validated self-report questionnaires were used to measure psychopathological symptoms, screening instruments may overestimate prevalence rates and cannot replace clinical diagnoses. Recruitment was limited to easily findable specialized clinics, self-help groups, and online communities, which may not be exhaustive. Given our commitment to participant anonymity, we were unable to trace the recruitment process for respondent-driven sampling. Exclusion of non-German speakers due to the questionnaire’s language further limits generalizability. Although online-based sampling yielded the largest number of participants in our study and therefore appeared to be the most effective recruitment strategy, the design of the study and the resulting data did not allow inferential statistical testing to formally compare recruitment effectiveness across the sampling methods. Consequently, these findings are limited to descriptive observations and should not be interpreted as evidence of statistically significant differences between the strategies. The unbalanced sample sizes were an inherent consequence of the study design, yet they also reduce representativeness, indicating that the findings should be interpreted as indicative rather than definitive for the broader rare disease population. Additionally, the distribution of continuous variables contained clear outliers, which may have skewed mean values and should therefore be interpreted with caution. Response categories with fewer than five observations were excluded from the statistical analyses due to insufficient statistical power and the risk of unreliable estimates, further limiting the interpretability of the results. Moreover, it is possible that, because of the small number of participants recruited via location-based sampling and respondent-driven sampling, the overall statistical power was not sufficient to detect all differences. A further limitation of this study is that recruitment was conducted without prior engagement with patient communities or patient representatives. This may have limited outreach and the number of participants reached. Future studies could consider involving patient communities more actively in the recruitment process, as such engagement may improve participation rates and the overall effectiveness of sampling strategies.

## Conclusion

In conclusion, our study indicates that in the field of rare diseases, online sampling appears to be the most resource-efficient and effective strategy for achieving larger sample sizes compared to respondent-driven and location-based sampling. Online sampling represents a promising approach for future studies, especially when seeking to maximize sample size within limited timeframes. However, it is important to consider the potential biases introduced by gender differences in social media usage patterns. Our study’s implementation of respondent-driven sampling faced considerable obstacles due to a combination of pandemic-induced changes in social structures and skepticism from key stakeholders. Overcoming these barriers will require a comprehensive strategy that includes effectively communicating the respondent-driven sampling methodology and considering the introduction of targeted incentives to boost participant engagement. Location-based sampling at specialized treatment centers may lead to higher completion rates but also failed to result in a large sample size in our study. Extending the study timeframe could be an option to improve effectiveness of this sampling strategy. Furthermore, building strong relationships with clinic staff and addressing their specific concerns may improve recruitment outcomes. However, the challenge of obtaining sufficiently large sample sizes to ensure representativeness remains a critical issue. Technological advancements such as the creation of patient registries are likely to improve the effectiveness of sampling in research on rare diseases. Ongoing innovation in these areas will be important for making research findings in this field more accurate and reliable.

## Electronic supplementary material

Below is the link to the electronic supplementary material.

Supplementary Material 1

## Data Availability

The datasets used and analyzed during the current study are available from the corresponding author on reasonable request in a fully anonymized form.
