# Supplementary material for: Optimizing recruitment in rare disease research: a cross-sectional online study evaluating sampling strategies for hard-to-reach populations
Source: Orphanet J Rare Dis. 2026 Jan 20;21:29. doi: 10.1186/s13023-025-04192-3 (PMC12837095; doi:10.1186/s13023-025-04192-3)
Supplement: Supplementary file 1 — Supplementary Material 1 [file 13023_2025_4192_MOESM1_ESM.docx]

**Additional files**

Additional file 1. Exact distribution of contacted instances supporting the recruitment.

| **Respondent-driven sampling^1^** | **Marfan syndrome** | **Huntington disease** | **Lysosomal storage**  **diseases** | **Total** |
| --- | --- | --- | --- | --- |
| contacted | 9 | 25 | 6 | 40 |
| received reply by | 5 | 1 | 6 | 12 |
| included in study | 6 | 0 | 5 | 11 |
| **Online-based sampling^2^** | **Marfan syndrome** | **Huntington disease** | **Lysosomal storage disorders** | **Total** |
| contacted | 1 | 3 | 7 | 11 |
| request accepted | 1 | 2 | 3 | 6 |
| upload via admin | 0 | 1 | 2 | 3 |
| included in study | 1 | 3 | 5 | 9 |
| **Location-based sampling^3^** | **Marfan syndrome** | **Huntington disease** | **Lysosomal storage disorders** | **Total** |
| contacted | 19 | 24 | 22 | 65 |
| received reply by | 5 | 15 | 12 | 32 |
| included in study | 5 | 6 | 3 | 14 |

Notes: ^1^ data refer to the number of seeds; ^2^ data refer to the number of groups, where study details were uploaded; ^3^ data refer to the number of outpatient clinics.
